# Development of a reproducible small intestinal microbiota model and its integration into the SHIME^®^-system, a dynamic *in vitro* gut model

**DOI:** 10.3389/fmicb.2022.1054061

**Published:** 2023-03-17

**Authors:** Stef Deyaert, Frédéric Moens, Walter Pirovano, Bartholomeus van den Bogert, Eline Suzanne Klaassens, Massimo Marzorati, Tom Van de Wiele, Michiel Kleerebezem, Pieter Van den Abbeele

**Affiliations:** ^1^ProDigest BV, Gent, Belgium; ^2^Baseclear, Leiden, Netherlands; ^3^Center of Microbial Ecology and Technology (CMET), Faculty of Bioscience Engineering, Ghent University, Gent, Belgium; ^4^Department of Animal Sciences, Wageningen University, Wageningen, Netherlands

**Keywords:** ileum, microbiota, *in vitro* model, short-chain fatty acid, small intestine, simulator of the human intestinal microbial ecosystem

## Abstract

The human gastrointestinal tract consists of different regions, each characterized by a distinct physiology, anatomy, and microbial community. While the colonic microbiota has received a lot of attention in recent research projects, little is known about the small intestinal microbiota and its interactions with ingested compounds, primarily due to the inaccessibility of this region *in vivo*. This study therefore aimed to develop and validate a dynamic, long-term simulation of the ileal microbiota using the SHIME^®^-technology. Essential parameters were identified and optimized from a screening experiment testing different inoculation strategies, nutritional media, and environmental parameters over an 18-day period. Subjecting a synthetic bacterial consortium to the selected conditions resulted in a stable microbiota that was representative in terms of abundance [8.81 ± 0.12 log (cells/ml)], composition and function. Indeed, the observed community mainly consisted of the genera *Streptococcus, Veillonella, Enterococcus, Lactobacillus*, and *Clostridium* (qPCR and 16S rRNA gene targeted Illumina sequencing), while nutrient administration boosted lactate production followed by cross-feeding interactions towards acetate and propionate. Furthermore, similarly as *in vivo*, bile salts were only partially deconjugated and only marginally converted into secondary bile salts. After confirming reproducibility of the small intestinal microbiota model, it was integrated into the established M-SHIME® where it further increased the compositional relevance of the colonic community. This long-term *in vitro* model provides a representative simulation of the ileal bacterial community, facilitating research of the ileum microbiota dynamics and activity when, for example, supplemented with microbial or diet components. Furthermore, integration of this present *in vitro* simulation increases the biological relevance of the current M-SHIME® technology.

## 1. Introduction

The human gastrointestinal tract (GIT) consists of distinct regions each comprising a unique physiology, anatomy, and microenvironment (Booijink et al., 2007; Donaldson et al., 2016; Ruan et al., 2020). The ecosystems of the stomach, small intestine and large intestine (or colon) together comprise hundreds of billions of bacteria, which are referred to as the gut microbiota (Bäckhed et al., 2005; Ley et al., 2006). This gut microbiota impacts human health through metabolizing undigested nutrients, producing health beneficial molecules, preventing pathogen colonization, and training the human immune system (Macfarlane and Macfarlane, 2011; Zoetendal et al., 2012; Arpaia et al., 2013; El Aidy et al., 2015; Marchesi et al., 2016). A key limitation for many studies that aim to investigate such interactions is the inaccessibility of the gastrointestinal tract, often resulting in the use of faecal samples which are only an approximation of what happens at the site of activity (Kastl et al., 2020). Therefore, a rather unexplored microbiota is the one that colonizes the small intestine. This microbiota is, nonetheless, of high interest since it is the first to encounter ingested food compounds and together with host physiology could have an important impact on stability and efficacy of ingested therapeutics (Zhang et al., 2018; van Kessel et al., 2019; van Kessel and El Aidy, 2019; Li et al., 2020).

The small intestine consists of the duodenum, jejunum, and ileum. Within these regions, microbial communities are shaped through distinct physiological parameters such as pH (Moore and Holdeman, 1974; Evans et al., 1988), transit time (Silvester et al., 1995), secretory products (Kitahara et al., 2004; Begley et al., 2005; O’May et al., 2005; Whitcomb and Lowe, 2007; Ridlon et al., 2014), and nutrient availability (Hao and Lee, 2004; reviewed in Booijink et al., 2007; Donaldson et al., 2016; Thursby and Juge, 2017). Furthermore, compared to the colon region, the small intestine comprises a higher surface area, thereby allowing intense interactions with host epithelial cells and the immune system through, e.g., Peyer’s patches (Stagg et al., 2004; Rakoff-Nahoum and Medzhitov, 2006). The intraluminal pH in the small intestine gradually increases from approximately 6 in the duodenum up to 7.4 in the terminal ileum (Evans et al., 1988; Booijink et al., 2007). Bile salts are typically present between 4 and 10 mM throughout the small intestine and can be toxic for bacteria through emulsification of their cell membranes (Islam et al., 2011; Kakiyama et al., 2013; Ridlon et al., 2014; Staley et al., 2017). Secretory products might comprise pancreatic enzymes among which trypsin, chymotrypsin, amylase and lipase are the most abundant ones (Whitcomb and Lowe, 2007). Furthermore, the small intestinal transit time is between 3 ± 1 h (Davis et al., 1986), which is an order of magnitude shorter as compared to the colonic residence time (39 ± 5 h; Arhan et al., 1981). Unlike the colon – where complex fibers are the main carbon source for bacteria – small intestinal microbes rather ferment simple sugars that are unabsorbed leftovers from digestion along the upper GIT (Borgström et al., 1957; Booijink et al., 2007, 2010; Zoetendal et al., 2012).

Bacterial densities along the small intestine are largely affected by region-specific physiological conditions. While abundances in the duodenum (10^3^–10^5^ cells per gram) and jejunum (10^4^–10^5^ cells per gram) are rather low due to the initial acidity coming from the stomach and high concentrations of bile salts and pancreatic enzymes, the more alkaline environment of the ileum comprises substantial numbers of bacteria (10^7^–10^8^ cells per gram; Booijink et al., 2007). However, microbial loads and diversity are relatively small compared to that of the colon (Booijink et al., 2007; Zoetendal et al., 2012; Thursby and Juge, 2017; Kastl et al., 2020). In terms of microbial composition, there are considerable inter-study variations (Thadepalli et al., 1979; Bouhnik et al., 1999; Riordan et al., 2001; Hayashi et al., 2005; Wang et al., 2005; Ivanov et al., 2008; Hartman et al., 2009; Booijink et al., 2010; Zoetendal et al., 2012; van den Bogert et al., 2013; El Aidy et al., 2015; Seekatz et al., 2019; Kastl et al., 2020), which are likely due to differences in test population (healthy adults vs. ileostomy patients vs. sudden death victims), sampling procedures (ileostomy effluent vs. autopsy vs. biopsy), sampling regions (lumen vs. mucus and proximal ileum vs. terminal ileum), and methodology to analyze the microbiota. This further adds to noise generated by strong inter-individual and temporal variation inherent to the ileal microbiota (Booijink et al., 2010; Zoetendal et al., 2012). While the ileal microbiota is often dominated by Firmicutes members, mainly from the genera *Streptococcus, Veillonella*, *Lactobacillus*, *Enterococcus*, and *Clostridium*, the small intestine from some individuals is distinct with high relative abundances of Proteobacteria and Actinobacteria, and specific genera such as *Fusobacterium*, *Faecalibacterium*, *Bacteroides*, *Ruminococcus*, *Blautia*, and *Prevotella* (Booijink et al., 2010; Zoetendal et al., 2012; van den Bogert et al., 2013; El Aidy et al., 2015; Cieplak et al., 2018; Seekatz et al., 2019). Furthermore, there seems to be consistency in metabolic functionality. Each elucidated ileal community comprises primary fermenters (e.g., *Streptococcus*, *Enterococcus*, and *Lactobacillus*) which metabolize simple carbohydrates into, e.g., lactate that supports growth of secondary fermenters (e.g., *Veillonella*). The latter consume lactate to produce short-chain fatty acids (SCFA; mainly acetate and propionate in the case of *Veillonella*) in a process termed cross-feeding (Foubert and Douglas, 1948; Egland et al., 2004; Hosseini et al., 2011; Scott et al., 2013; Kastl et al., 2020).

Due to interindividual variations, longitudinal dynamics, and inaccessibility of the ileal microbiota *in vivo,* development of robust *in vitro* models of the ileal microbiota could be useful to better understand the processes that drive the small intestinal microbiota (Booijink et al., 2010). Most of the currently available *in vitro* models of the small intestine focus on host physiology and lack representation of its colonizing microbiota (Guerra et al., 2012). In contrast, models that incorporate the ileal microbiota are rather short-term and include, e.g., dosing a mixture of ileum-specific bacterial strains which are then immediately investigated, thus being unsure whether they thrive well in the applied environment (Cieplak et al., 2018). Other models have also tried to simulate the ileal microbiota over longer periods of time, but the emerged communities appeared to resemble more colon-like communities with high abundances of either Bacteroidetes (Stolaki et al., 2019) or Enterobacteriaceae (Roussel et al., 2020), or showed strong temporal fluctuations (Roussel et al., 2020). Finally, miniaturized *in vitro* models such as gut-on-a-chip have been designed that can even recreate the anoxic–oxic interface defining the mucosal-bacteria microenvironment (Bein et al., 2018). However, establishing a stable gut microbiota in such a system for long-term studies is nearly impossible due to the very small handling volumes. Although these models present their own advantages, a dynamic, long-term *in vitro* model of the ileal microbial community would enable more mechanistic research, such as studying changes in microbial interactions or metabolic shifts upon administration of a compound of interest. To the best of our knowledge, there is no such model available yet.

Therefore, the aim of the current study was to develop a novel *in vitro* model which – with properly optimized environmental conditions – supports a microbiota that is representative in function and composition to the *in vivo* ileal microbiota during a period of multiple weeks. A second goal was to integrate this small intestinal microbiota model in the established Mucosal Simulator of the Human Intestinal Microbial Ecosystem (M-SHIME®) which already allows biorelevant simulation of the colon microbiota (Molly et al., 1993; Van den Abbeele et al., 2012).

## 2. Materials and methods

### 2.1. Chemicals

Chemicals were obtained from Alfa Aesar (Kandel, Germany), Carl Roth (Karlsruhe, Germany), Chem-Lab (Zedelgem, Belgium), Keyser & Mackay (Brussels, Belgium), Merck (Overijse, Belgium), Oxoid (Merelbeke, Belgium), Sigma-Aldrich (Overijse, Belgium), and VWR (Leuven, Belgium), as elaborated below.

### 2.2. Microbial inocula

During the present study, several inoculation strategies were compared. This involved the use of faecal inocula from healthy human adults collected and prepared as a 20% (w/v) solution of the fecal sample in anaerobic phosphate buffer as previously described (Molly et al., 1993), ileostomy effluents and a synthetic consortium comprising 12 bacterial species (Table 1). Samples derived from human donors were collected according to the ethical approval of the University Hospital Ghent with reference number B670201836585.

**Table 1:** Bacterial strains, their source and applied growth conditions.

Species	Strain source	Growth medium	Growth condition
*Streptococcus bovis*	LMG 8518	TSB	Aerobic
*Streptococcus intermedius*	LMG 17840	TSB	Aerobic
*Ligilactobacillus salivarius*	LMG 9477	MRS + 0.1% Tween®80	Aerobic
*Limosilactobacillus reuteri*	LMG 9213	MRS + 0.1% Tween®80	Aerobic
*Enterococcus faecalis*	LMG 7937	MRS	Aerobic
*Enterococcus faecium*	DSM 20477	MRS	Aerobic
*Veillonella parvula*	LMG 30945	Veillonella medium	Anaerobic
*Veilonella dispar*	DSM 20735	Veillonella medium	Anaerobic
*Blautia obeum*	DSM 25238	RCM + 0.01% mucin	Anaerobic
*Faecalibacterium prausnitzii*	DSM 17677	RCM	Anaerobic
*Clostridium nexile*	LMG 28906	RCM	Anaerobic
*Prevotella melaninogenica*	LMG 28911	RCM	Anaerobic

All species were incubated at 37°C. Species grown under aerobic conditions were shaken continuously at 90RPM, while species grown under anaerobic conditions were grown in a static fashion. Grown cultures were harvested during their exponential growth phase. TSB, tryptic soy broth (Oxoid); MRS, de Man, Rogosa and Sharpe broth (Oxoid); RCM, reinforced clostridial medium (Oxoid); Veillonella medium was prepared according to BCC/LMG medium 1,107 (BCC/LMG, Ghent, Belgium).

### 2.3. Preparation of glycerol stocks of strains selected for a small intestinal consortium

Glycerol stocks were prepared for each strain separately. Grown cultures were harvested during exponential growth and 0.5 ml culture was mixed with 0.5 ml glycerol-based cryoprotectant (50% v/v glycerol (86%) [Merck], 0.5 g L^−1^ l-cysteine-HCl [Merck], 10 g L^−1^ trehalose [Sigma-Aldrich], 3 g L^−1^ tryptic soy broth (TSB) [Oxoid], 18 g L^−1^ NaCl [Chem-Lab], 1 ml L^−1^ resazurin [Alfa Aesar] stock solution (2% w/v) and 50% (v/v) dH_2_O). For strict anaerobic strains, the glycerol-based cryoprotectant was boiled for 1′ to reduce oxygen content prior to autoclaving. Glycerol stocks were stored at −80°C until usage.

### 2.4. *In vitro* M-SHIME^®^ technology

All experiments were performed through modification of the M-SHIME® technology previously described by Van den Abbeele et al. (2012). Setups were autoclaved prior to inoculation to assure sterility of the vessels. Furthermore, reactors were continuously homogenized through stirring, maintained at 37°C, and kept anaerobically by flushing with N_2_ for 15 min, three times per day.

#### 2.4.1. Nutritional media

Ileal nutritional medium (100%) was prepared by sterile addition (5% v/v) of a filter-sterilized stock solution containing (L^−1^) NaHCO_3_ (50 g; Chem-Lab), NaH_2_PO_4_ (10 g; VWR), K_2_HPO_4_ (10 g; Chem-Lab), MgSO_4_.7H_2_O (0.9 g; Chem-Lab), MnCl_2_.4H_2_O (0.5 g; VWR International Europe BVBA), CaCl_2_.2H_2_O (0.9 g; VWR), FeSO_4_.7H_2_O (0.05 g; Sigma-Aldrich), ZnSO_4_.7H_2_O (0.05 g; Sigma-Aldrich), hemin (0.05 g; Sigma-Aldrich), glucose (12 g; Merck), fructose (12 g; Sigma-Aldrich), sucrose (12 g; Sigma-Aldrich), maltose (12 g; Carl Roth), lactose (12 g; Oxoid), mannose (7.5 g; Carl Roth), and galactose (7.5 g; Sigma-Aldrich) to an autoclaved medium comprising (L^−1^) bile salts (1.9 g; BD Bioscience, Erembodegem, Belgium), special peptone (0.7 g; Oxoid), yeast extract (2.2 g; Oxoid), mucin (2.2 g; Carl Roth), l-cysteine-HCl (0.4 g; AXO Industry SA, Wavre, Belgium) and Tween®80 (1.1 ml; Sigma-Aldrich). Ileal nutritional medium (30%) was equal to ileal nutritional medium (100%) apart from a 70% reduction in simple sugars, special peptone, yeast extract, mucin and cysteine-HCl. Autoclaved colonic nutritional medium (set at pH 2 with 37% HCl) was added to proximal colon (PC) reactors and comprised (in g L^−1^) arabinogalactan (1.2; Keyser & Mackay), pectin (2.0; Keyser & Mackay), xylan (0.5; Carl Roth), starch (4.0; Carl Roth), glucose (0.4; Merck), yeast extract (3.0; Oxoid), peptone (1.0; Oxoid), mucin (2.0; Carl Roth), and l-cysteine-HCl (0.5; Merck). When preceded by an ileum reactor, PC reactors received 50 ml of fiber solution instead of nutritional colon medium. Fiber solution contained (in g L^−1^) arabinogalactan (3.4; Keyser & Mackay), pectin (5.6; Keyser & Mackay), xylan (1.4; Carl Roth), starch (11.2; Carl Roth), glucose (1.12; Merck), peptone (2.1; Oxoid), yeast extract (6.3; Oxoid), mucin (4.2; Carl Roth) and l-cysteine-HCl (1.0; Merck). This resulted in the same influx of nutrients in the proximal colon, independent from the incorporation of an ileum reactor.

#### 2.4.2. Simulation of mucosal microbiota

Mucin-alginate beads were used for small intestinal simulations and prepared by dripping a 5×-boiled mucin-alginate solution [50 g L^−1^ mucin (Carl Roth), 12 g L^−1^ agar (VWR), 12 g L^−1^ alginate (Carl Roth) and 2.22 ml L^−1^ 10 M NaOH (Chem-Lab)] into crosslinking solution containing 7.6 g L^−1^ CaCl_2_.2H_2_O (VWR). This approach was implemented as the small alginate beads allowed for sterile sampling of colonized beads and addition of fresh beads *via* a 50 ml-syringe with catheter tip (Novolab) connected to an inlet port. Sterility of such handlings was a prerequisite as one worked with a synthetic consortium in multiple ileal simulations. In contrast, for the colonic microbiota, the conventional approach using mucin-covered microcosms was used as previously described by Van den Abbeele et al. (2012). A buffer comprising (g L^−1^) K_2_HPO_4_ (8.8; Chem-Lab) and KH_2_PO_4_ (6.8; Chem-Lab) was used to rinse luminal content from mucosal samples. Half of the mucus-alginate beads and mucin-covered microcosms were replaced every 2 days.

#### 2.4.3. Experimental designs

##### 2.4.3.1. Screening experiment

**Figure 1:**
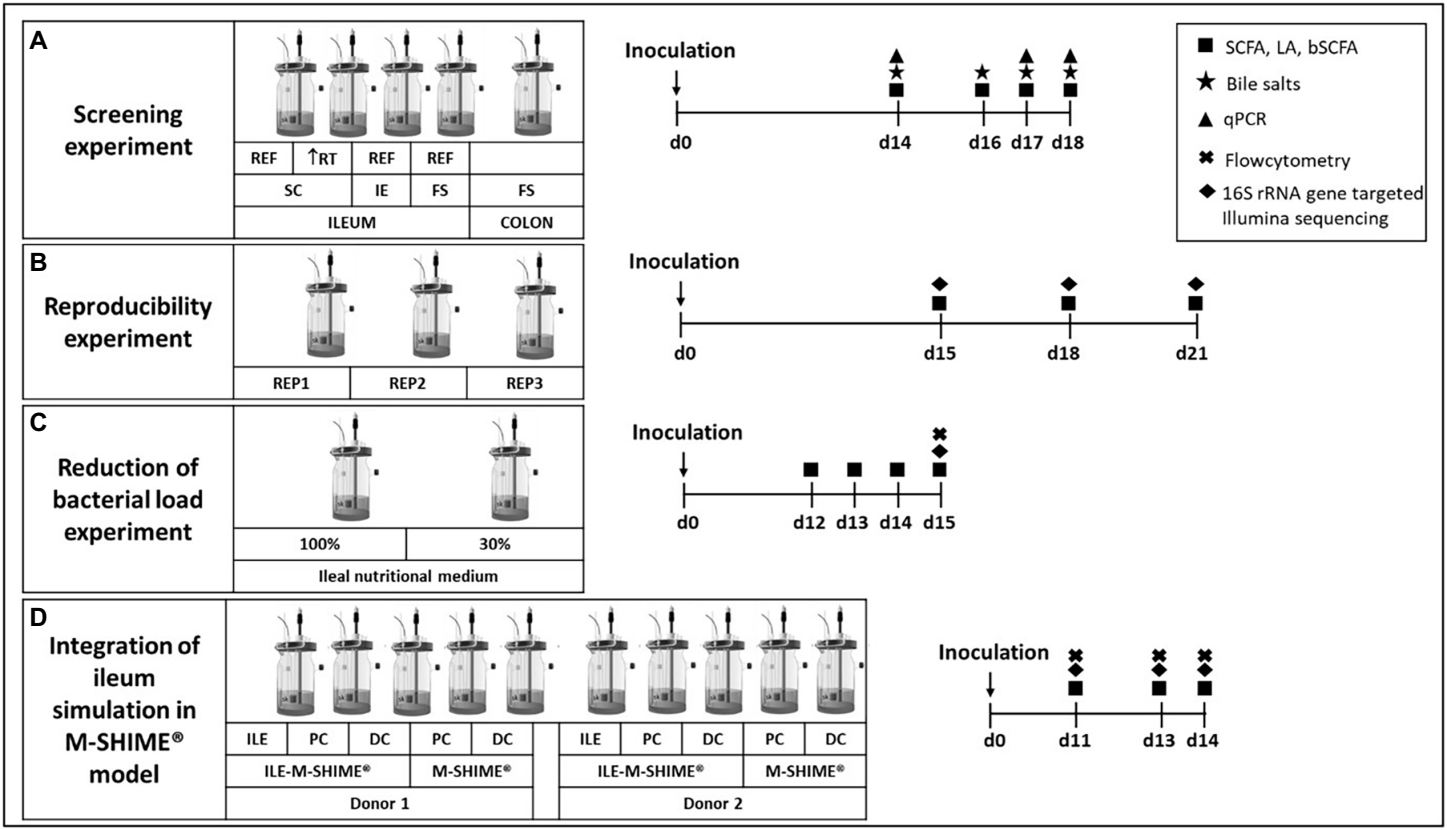
Experimental design for **(A)** screening experiment to compare different inoculation strategies (synthetic consortium, ileostomy effluent, or faecal slurry) under reference conditions (RT = 4 h) or upon doubled retention time (RT = 8 h) and a control colon simulation; **(B)** reproducibility experiment to confirm reproducibility of the synthetic consortium inoculation under reference conditions as selected from screening experiment; **(C)** reduction of bacterial load experiment to evaluate effect on bacterial levels upon feeding the simulation with a nutritional medium comprising reduced sugar levels; and **(D)** the validation experiment for integrating the ileum simulation in the M-SHIME^®^ model. Numbers indicate the number of days after inoculation of the model. REF = reference conditions; RT = retention time; IE = ileostomy effluent; FS = faecal slurry.

In a first experiment, different conditions were compared for their potential to maintain an ileal-representative microbiota (Figure 1A). For this purpose, the SHIME® (ProDigest, Ghent, Belgium and Ghent University, Ghent, Belgium) was configured for comparison of four parallel ileal and one colon simulation. The ileal simulations differed in inoculation strategy, i.e., synthetic consortium (vessel 1 and 2; mixture containing equal volumes of glycerol stocks of 12 strains – Table 1), ileostomy effluent (vessel 3; 5 ml of a 1:5 dilution) and faecal slurry (vessel 4; 5 ml of a 1:5 dilution). Moreover, while the standard retention time of ileal simulations was 4 h, an additional condition with increased retention time of 8 h was evaluated (reactor 2). At the start, ileal reactors were filled with 100 ml ileal nutritional medium (100%) and 5 g sterile mucin-alginate beads, while the colon vessel was filled with 500 ml of colon nutritional medium and 32 mucin-covered microcosms (Van den Abbeele et al., 2012). The pH of ileal vessels was controlled at 7.05–7.35, while the pH of the colon vessel was set at 6.15–6.40 (and a colonic retention time of 20 h was mimicked). Fresh nutritional medium was administered three times per day. Samples were collected on days 14, 16, 17 and 18 for analysis of SCFA, lactate, branched chain fatty acids (BCFA), bile salts and microbial composition *via* qPCR. Additionally, on day 14, sampling was performed at 0, 0.5, 1, 2, 4, and 8 h after entrance of fresh nutritional medium to elucidate the kinetic metabolic profile in terms of SCFA and lactate production.

##### 2.4.3.2. Reproducibility experiment

During a second experiment, observations from the screening experiment were validated by repeating test conditions of vessel 1 in triplicate (Figure 1B). Samples were collected on days 15, 18, and 21 for analysis of SCFA, lactate, BCFA and 16S rRNA gene targeted Illumina sequencing.

##### 2.4.3.3. Reduction of bacterial load

A third experiment aimed to reduce bacterial densities in the ileum simulation (Figure 1C). Therefore, two ileum simulations were performed in parallel. Parameters for both vessels were identical to the test conditions of vessel 1 from the screening experiment and vessels 1–3 from the reproducibility experiment, with the exception that the second vessel was fed with 30% ileal nutritional medium. Samples were collected on days 12, 13, 14, and 15 for analysis of SCFA, lactate and BCFA. On the final day, microbial composition was analyzed *via* 16S rRNA gene targeted Illumina sequencing coupled with flow cytometry.

##### 2.4.3.4. Integration of ileum simulation in M-SHIME^®^ model

The final experiment evaluated integration of the ileum simulation in the M-SHIME® model as described by Šuligoj et al. (2020; Figure 1D). The configuration was adapted to evaluate the impact of a preceding ileum onto the proximal colon (PC) and distal colon (DC) simulations. For this, the setup comprised of a first arm where the PC and DC were preceded by an ileum simulation (i.e., ILE-M-SHIME®), while a second arm comprised of a PC and DC without preceding ileum (i.e., M-SHIME®), and this for two donors. The ileum reactors were simulated under conditions identical to the reactor with decreased bacterial load, thus receiving ileal nutritional medium 30%. Regarding the colonic simulations, the pH was controlled between 5.70–5.90 for PC vessels, and between 6.60–6.90 for DC vessels. Pumping times were set so that retention times in the PC and DC vessels were 20 and 32 h, respectively. For ILE-M-SHIME® units, the proximal colons received both excess volume from the ileum vessels (approximately 150 ml) and 50 ml fiber solution. Instead, in M-SHIME® units, the proximal colon vessels received a mixture of colon nutritional medium, and pancreatic and bile liquid from the stomach vessel as described previously (Molly et al., 1993; Possemiers et al., 2004; Van den Abbeele et al., 2012, 2013; Ghyselinck et al., 2020; Šuligoj et al., 2020). Samples were collected on days 11, 13, and 14 for analysis of SCFA, lactate, BCFA and 16S rRNA gene targeted Illumina sequencing coupled with flow cytometry.

### 2.5. Metabolic activity analysis

Quantification of SCFAs, including acetate, propionate, butyrate, and branched SCFAs (BSCFA; i.e., isobutyrate, isovalerate, and isocaproate) was performed as previously reported by Ghyselinck et al. (2020). Lactate concentrations were determined on supernatant aliquots (centrifuged for 5 min at 7,690 × *g*) using a commercially available enzymatic assay kit according to the manufacturer’s instructions. For the screening experiment and reproducibility experiment, the enzymatic assay kit of Roche was used (Roche Diagnostics, Machelen, Belgium) while the R-Biopharm kit was used for lactate quantification of the optimization experiment and incorporation experiment (R-Biopharm, Darmstadt, Germany). Concentrations of the bile salts taurocholic acid (TCA), glycocholic acid (GCA), taurodeoxycholic acid (TDCA), and glycodeoxycholic acid (GDCA) were determined through reversed-phase high pressure liquid chromatography (RP-HPLC; Hitachi Chromaster, Hitachi, Brussels, Belgium) with a diode array detector (DAD; VWR), using a reversed-phase C18 column (Hydro-RP, 4 μm, 80 Å, 250 × 4.6 mm, Synergi, Phenomenex BV, Utrecht, The Netherlands). The mobile phase at a flow rate of 0.7 ml/min consisted of acetonitrile (eluent A; VWR), and ultrapure HPLC-grade H_2_O at a pH of 2 (eluent B; VWR), with the following gradient: 0.0 min, 30% A and 70% B; 70 min, 90% A and 10% B; 71 min, 30% A and 70% B; and 75 min, 30% A and 70% B. The bile salts were detected through DAD at a wavelength of 210 nm. Sample preparation involved centrifugation of samples at 7,690 × *g* for 5 min before storage at −20°C. Afterwards, 500 μl of supernatant was added to 500 μl of methanol following centrifugation at 7,690 × *g* for 5 min. Finally, the supernatant was filtered through a PTFE filter (0.2 μm; VWR) prior to injection (50 μl) onto the column. Quantification of samples was performed by using external standards.

### 2.6. Microbial community analysis

For bacterial community analysis, DNA was extracted as previously described by Boon et al. with some minor modifications (Boon et al., 2003). Homogenization was performed using a Beadblaster device (Benchmark Scientific, Edison, NJ, United States), which was conducted twice for 40 s at 6.00 m/s with a cooling period of 5 min between shakings. As an exception, DNA from samples of the final experiment was extracted *via* the ZymoBIOMICS 96 MagBead DNA Kit (Zymo Research, Irvine, CA, United States) at the KingFisher Flex Purification System (Thermo Fischer Scientific, Waltham, MA, United States) according to the manufacturer’s instructions. Luminal DNA originated from pellets obtained from 1 ml sample, while mucosal DNA was extracted from 0.25 g mucin alginate agar (ileum) or mucin agar (colon).

Targeted microbial quantification was determined through quantitative polymerase chain reaction (qPCR) on a QuantStudio 5 Real-Time PCR system (Applied Biosystems, Forster City, CA, United States). Standard curves were generated from a 10-fold dilution series ranging from 10^6^ gene copies/μl to 10 gene copies/μl. Except for qPCRs targeting Streptococcaceae and Veillonellaceae – which used PCR product –, plasmid DNA was used to generate standard curves. Each sample was run in technical triplicate and outliers were removed when standard deviation between replicates exceeded 0.5. qPCRs for following groups were performed as previously described: *Lactobacillus* spp. (Furet et al., 2009), *Bifidobacterium* spp. and *Eubacterium rectale*/*Clostridium coccoides* (Rinttilä et al., 2004), Bacteroidetes (Guo et al., 2008), *Faecalibacterium prausnitzii* (Sokol et al., 2009), Veillonellaceae (Rinttilä et al., 2004), Enterobacteriaceae (Nakano et al., 2003), Streptococcaceae (van den Bogert et al., 2011), Enterococcaceae (Rinttilä et al., 2004), and *Akkermansia muciniphila* (Collado et al., 2007). Illumina 16S rRNA gene amplicon libraries were generated and sequenced at BaseClear BV. In short, barcoded amplicons from the V3-V4 region of 16S rRNA genes were generated using a 2-step PCR. 10 ng genomic (g)DNA was used as template for the first PCR with a total volume of 50 μl using the 341F (5′-CCTACGGGNGGCWGCAG-3′) and the 785R (5′-GACTACHVGGGTATCTAATCC-3′) primers appended with Illumina adaptor sequences. PCR products were purified, and the sizes of the PCR products were checked on Fragment analyzer (Agilent, Santa Clara, CA, United States) and quantified by fluorometric analysis. Purified PCR products were used for the second PCR in combination with sample-specific barcoded primers (Nextera XT index kit; Illumina, San Diego, CA, United States). Subsequently, PCR products were purified, checked on a Fragment analyzer (Agilent) and quantified, followed by multiplexing, clustering, and sequencing on an Illumina MiSeq with the paired-end (2×) 300 bp protocol and indexing. The sequencing run was analyzed with the Illumina CASAVA pipeline (v1.8.3) with demultiplexing based on sample-specific barcodes. The raw sequencing data was processed by removal of sequence reads of too low quality (only “passing filter” reads were selected) and discard of reads containing adaptor sequences or PhiX control with an in-house filtering protocol. A quality assessment on remaining reads was performed using the FASTQC quality control tool version 0.10.0. For data processing, Illumina-paired reads were merged into single reads (pseudoreads) through sequence overlap, after removal of the forward and reverse primers. Chimeric pseudoreads were removed and remaining reads were aligned to the RDP 16S gene databases. Based on the alignment scores of the pseudoreads, the taxonomic depth of the lineage is based on the identity threshold of the rank; Species 99%, Genus 97%, Family 95%, Order 90%, Class 85%, Phylum 80%.

Total bacterial cells were quantified *via* flow cytometry to convert proportional data obtained *via* 16S rRNA gene targeted Illumina sequencing to quantitative data (multiplication). Samples were 1:1-diluted in cryoprotectant and stored at −80°C until analysis according to Hoefman et al. (2012). Upon staining with 0.01 mM SYTO24 (Life Technologies Europe NV, Merelbeke, Belgium) at room temperature for 15 min in the dark, samples were analyzed on a BD Facsverse (BDBiosciences, Merelbeke, Belgium) using the high flowrate setting and bacteria were separated from medium debris and signal noise by applying a threshold level of 200 on the SYTO channel. Flow cytometry data were analyzed using FlowJo, version 10.5.0.

### 2.7. Statistics

Statistically significant differences between acetate, propionate, butyrate, lactate, and bile salt concentrations were determined for each experiment separately. Significant differences on bacterial abundances were determined on absolute levels for luminal samples and relative levels for mucosal samples for the respective experiments. Statistical analysis was performed in Excel 2011 (Microsoft, Redmond, WA, United States). Two-sided t-tests were applied for comparisons between different vessels during statistical analysis of all experiments. Benjamini-Hochberg false discovery rate (FDR) was applied (with FDR = 0.05) to correct for multiplicity issues as described previously (Lee and Lee, 2018). Principal component analysis (PCA) was performed with CLUSTVIS (biit.cs.ut.ee/clustvis/) using the proportional 16S rRNA gene targeted Illumina sequencing data of the 15 most abundant families in the lumen across all simulated communities supplemented with taxonomic families representing consortium genera but not being part of the top 15 most abundant families.

## 3. Results

### 3.1. Distinct environmental conditions strongly impacted bacterial composition and activity

During an initial screening experiment, four test conditions were evaluated in parallel and compared to a control colon simulation. Reactors 1 and 2 were inoculated with a synthetic consortium, while reactors 3 and 4 were inoculated with ileostomy effluent and faecal sample, respectively. Reactor 2 was distinguished from reactor 1 in that it was subjected to a longer retention time (8 h instead of 4 h). Both the inoculation strategies and retention time affected microbial colonization during the ileum simulations in terms of microbial composition and activity.

First, the validity of a qPCR panel in which 10 taxonomic groups were targeted was evaluated using an ileostomy effluent and faecal sample (Supplementary Table S1). This confirmed the presence of distinct microbial community compositions in both samples, in correspondence with literature, i.e., enrichment of Veillonellaceae, Streptococcaceae, and Enterococcaceae (and Lactobacillaceae depending on the intake of probiotics) in ileostomy samples in contrast to higher levels of Enterobacteriaceae, Akkermansiaceae, Bifidobacteriaceae, Bacteroidetes, Lachnospiraceae, and *F. prausnitzii* in the faecal sample (Supplementary Table S1), thus confirming that the qPCR panel allowed to make representative statements on whether a microbial community is more ileum or colon-like.

This allowed to conclude that the steady-state communities of the screening experiments upon 2 weeks of colonization were largely affected by the inoculation strategy, with more ileum-like communities being obtained upon inoculation with the synthetic consortium. Indeed, the latter (upon applying a transit time of 4 h) resulted in a community dominated by Veillonellaceae [8.73 ± log(copies/ml)], Streptococcaceae [8.94 log(copies/ml)], Enterococcaceae [9.16 log(copies/ml)] and to a lesser extent, Lactobacillaceae [6.81 log(copies/ml)] (Table 2). Strikingly, incubating the same consortium at increased retention time (8 h) induced dominance by Enterococcaceae [9.52 log(copies/ml)] and Lactobacillaceae [7.53 log(copies/ml)] at the expense of Veillonellaceae [3.50 log(copies/ml)] and Streptococcaceae [3.46 log(copies/ml)]. Further, inoculation with ileostomy effluent or faecal slurry increased abundances of colon-associated taxonomy groups such as Enterobacteriaceae [6.79 and 7.15 log(copies/ml), respectively], Akkermansiaceae [4.45 and 4.38 log(copies/ml), respectively], Bifidobacteriaceae [8.38 and 8.40 log(copies/ml), respectively], Bacteroidetes [7.83 and 7.96 log(copies/ml), respectively], *C. coccoides/E. rectale* [8.64 and 8.90 log(copies/ml)], and *F. prausnitzii* [5.91 and 7.41 log(copies/ml)]. The latter more closely resembled the stable community composition in the control colon vessel.

**Table 2:** Average absolute levels [^10^log(16S rRNA copies/ml)] of 10 different taxonomic groups (as determined with group-specific qPCR protocols) that, according to literature, colonize the small intestine and/or colon, when incubated in reactors under ileal or colonic conditions.

Region of colonization according to literature	Microbial group	Ileum				Colon
		Consortium		Ileostomy effluent	Faecal slurry	Faecal slurry
		REF	↑ RT			
Small intestine	Veillonellaceae	**8.73**^**a**^	3.50^c^	3.97^b,c^	<LOQ^c^	4.84^b^
	Streptococcaceae	**8.94**^**a**^	3.46^e^	7.81^b^	6.94^c^	5.19^d^
	Enterococcaceae	**9.16**^**a**^	**9.52**^**a**^	7.76^b^	7.03^b^	5.42^c^
	Lactobacillaceae	6.81^b^	**7.53**^**a**^	3.49^c^	3.51^c^	3.71^c^
Small intestine – colon	Enterobacteriaceae	<LOQ^c^	<LOQ^c^	**6.79**^**a,b**^	**7.15**^**a**^	6.31^b^
Colon	Akkermansiaceae	< LOQ^c^	<LOQ^c^	**4.45**^**a,b**^	**4.38**^**a**^	3.93^b^
	Bifidobacteriaceae	<LOQ^c^	<LOQ^c^	**8.38**^**a**^	**8.40**^**a,b**^	7.65^b^
	Bacteroidetes	4.70^c^	<LOQ^c^	7.83^b^	7.96^b^	**8.42**^**a**^
	*C. coccoides*/*E. rectale*	<LOQ^b^	3.47^b^	**8.64**^**a**^	**8.90**^**a**^	**8.76**^**a**^
	*F. prausnitzii*	<LOQ^c^	<LOQ^c^	5.91^b^	**7.41**^**a**^	5.12^b^

While the colonic simulations started with a faecal inoculation, the ileal simulations involved inoculation of a consortium, ileostomy effluent or faecal slurry. While the standard residence time (RT) was 4 h for the ileal simulations, one condition involved an increased residence time of 8 h. Values that are significantly different from one another are indicated with a different letter (a, b, c, d, e). Values for the condition(s) that resulted in the highest levels measured for a given taxonomic group are indicated in bold. LOQ = 3.31 ^10^log(cells/ml). REF, reference conditions.

**Figure 2:**
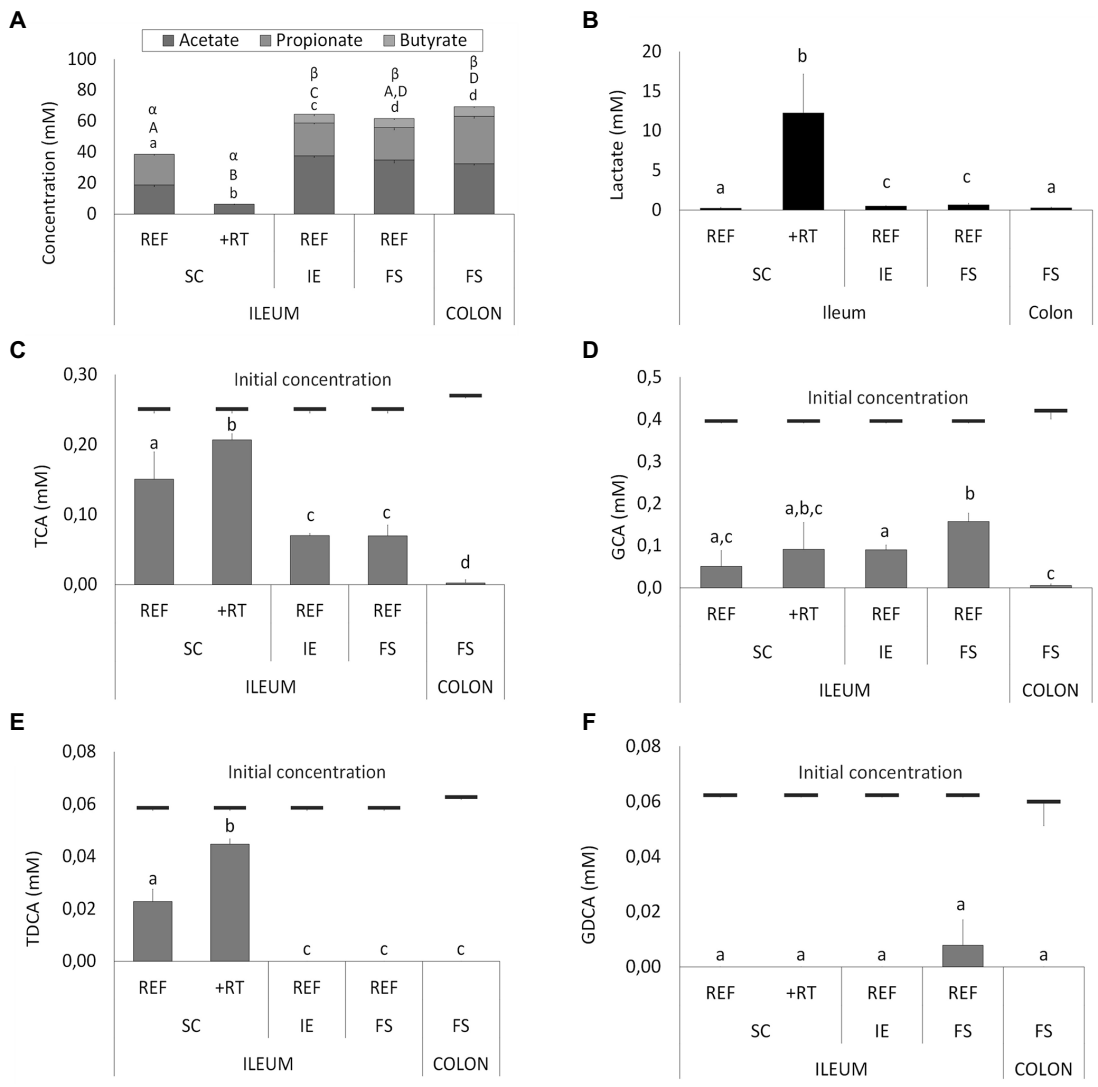
**(A)** Average short-chain fatty acids (SCFA) concentrations (mM) from days 14, 16, 17, and 18 in different ileum simulation test conditions. Values that significantly differ from each other are indicated with different letters (a, b, c, d for acetate; A, B, C, D for propionate, and α, β for butyrate). **(B)** Average lactate concentrations (mM) in different test conditions. Values that significantly differ from each other are indicated with different letters (a, b, c). **(C–F)** Average bile salt concentrations in different test conditions. Red bars indicate the initial concentration of the respective bile salt in the simulation upon administration of fresh nutritional medium. For ileum test conditions, either reference conditions (REF) or increased retention time (+RT) was tested with different inoculation strategies (SC, IE, and FS). For the colon, parameters from M-SHIME® technology were applied. SC = synthetic consortium; IE = ileostomy effluent; FS = faecal slurry; TCA = taurocholic acid; GCA = glycocholic acid; TDCA = taurodeoxycholic acid; GDCA = glycodeoxycholic acid.

**Figure 3:**
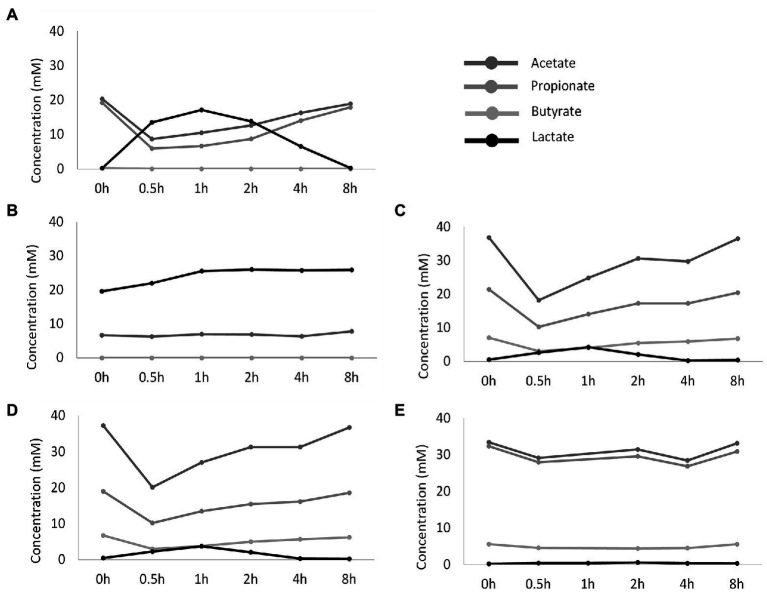
Temporal profile of SCFA and lactate concentrations (mM) upon administration of fresh nutritional medium to different test conditions comprising a steady-state community: **(A)** reference conditions inoculated with synthetic consortium; **(B)** doubled retention time inoculated with synthetic consortium; **(C)** reference conditions inoculated with ileostomy effluent; **(D)** reference conditions inoculated with faecal slurry; **(E)** M-SHIME® proximal colon simulation.

These drastic differences in microbiota composition significantly affected microbial activity. In a first aspect, inoculation with the synthetic consortium significantly lowered total SCFA levels in the reference condition (38.6 ± 1.1 mM) and even further upon increased retention time (6.5 ± 0.2 mM) as compared to the reference conditions inoculated with ileostomy effluent and faecal slurry (64.5 ± 1.1 mM and 61.7 ± 2.3 mM, respectively), while total SCFA levels were the highest in the control colon (69.4 ± 1.9 mM; Figure 2A). In a second aspect, steady-state lactate levels were similarly low among all reference conditions and control colon, which might indicate efficient cross-feeding of lactate to, e.g., propionate and/or butyrate. In contrast, the prolonged retention time significantly increased lactate, thereby suggesting impaired cross-feeding (Figure 2B). In addition, kinetic sampling during 8 h upon receiving fresh nutritional medium on day 14 of the experiment revealed an initial boost in lactate production followed by lactate consumption and a concomitant production of acetate and propionate for the reference consortium condition (Figure 3). Although to a lesser extent, this kinetic profile was also observed for the reference conditions inoculated with ileostomy effluent and faecal slurry. In contrast, entrance of nutritional medium into the condition with extended retention time increased lactate production but lacked the consequential consumption of lactate and concomitant acetate and propionate production (corresponding with the low Veillonellaceae levels upon increased retention time). The control colon showed a modest increase in acetate, propionate, and butyrate concentrations over time while no increase in lactate level was observed.

Furthermore, different conditions affected bile salt metabolism both between the test conditions and as compared to the control colon (Figures 2C–F). The latter showed complete deconjugation of TCA, GCA, TDCA, and GDCA, while said bile salts were only partially deconjugated in the ileal vessels. Lowest bile salt conversions were observed upon inoculation with the synthetic consortium, and more specific, in combination with an increased retention time.

Overall, from all tested conditions, the synthetic consortium inoculation under reference conditions was selected as most closely representing the *in vivo* ileal community (Booijink et al., 2010; Zoetendal et al., 2012; El Aidy et al., 2015; Cieplak et al., 2018; Seekatz et al., 2019).

### 3.2. The selected condition allows establishment of a stable and reproducible community

Next, the selected reference condition from the screening experiment was performed in triplicate to validate reproducibility of the simulation.

**Figure 4:**
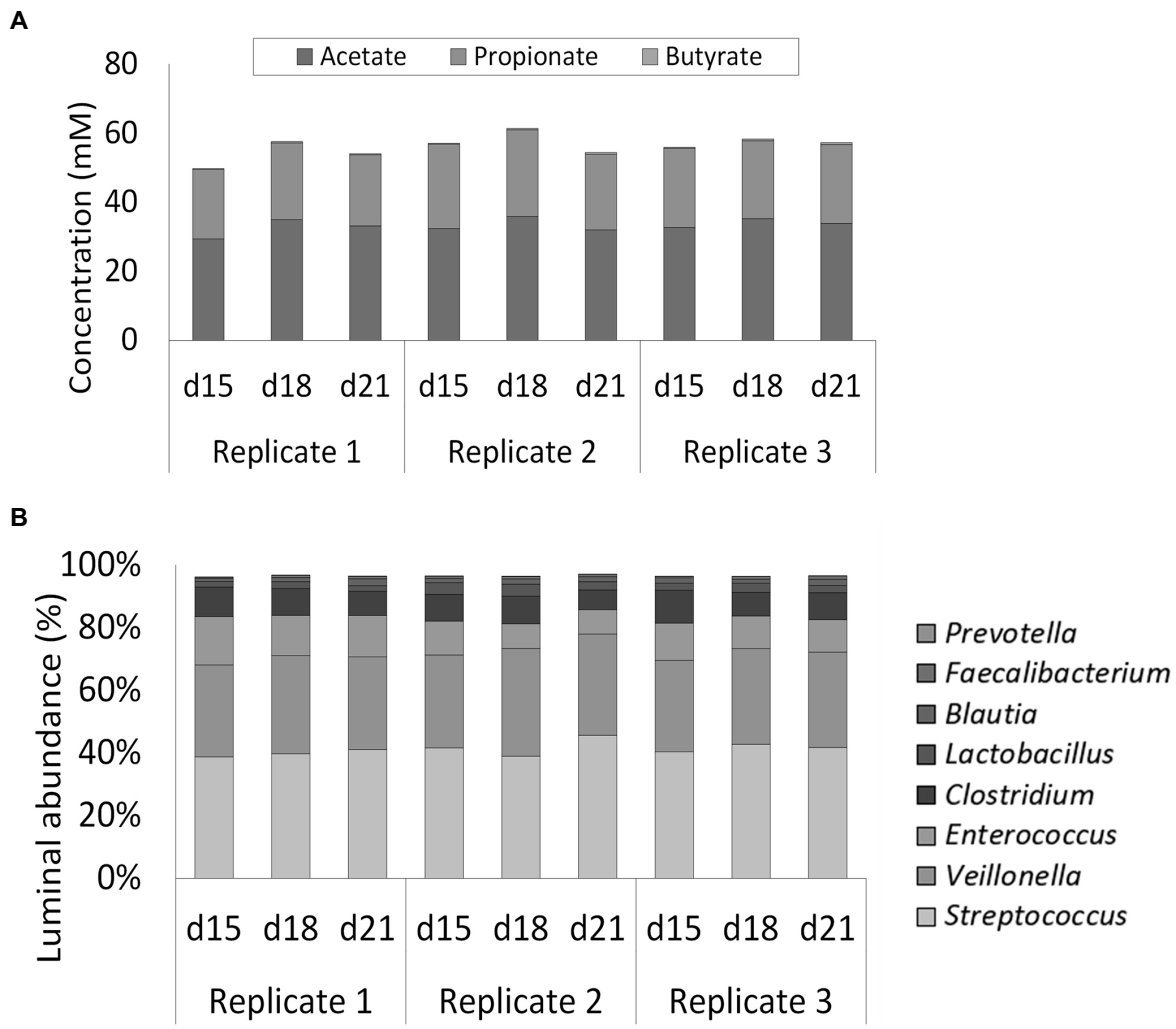
**(A)** Short-chain fatty acids (SCFA) concentrations (mM) in three replicate ileum simulations at three different timepoints (days 15, 18, and 21 after inoculation). All replicates represented the reference conditions inoculated with synthetic consortium. **(B)** Average genus level relative microbial community composition of three ileum simulation replicates incubated under reference conditions after inoculation with synthetic consortium at three different timepoints (days 15, 18, and 21 after inoculation). Genera included in the consortium account for nearly 100% of the steady-state community. The reference *Lactobacillus* is considered to include both the genera *Limosilactobacillus* and *Ligolactobacillus*.

Luminal SCFA profiles were stable over time (87, 86, and 88% for replicates 1, 2, and 3, respectively) and highly reproducible (95%) across the multiple replicate vessels of the selected condition (Figure 4A). Lactic acid levels were 1.40 ± 0.69 mM on average and did not differ significantly between replicate simulations (data not shown). 16S rRNA gene targeted Illumina sequencing demonstrated a nearly identical community composition within the replicate vessels at different timepoints, thereby confirming both stability and reproducibility of the bacterial community (Figure 4B). Luminal communities were dominated by the genera *Streptococcus* (41.2 ± 2.5%), *Veillonella* (30.7 ± 2.0%), *Enterococcus* (11.2 ± 2.7%), and *Clostridium* (8.4 ± 1.3%). The genera *Lactobacillus* (2.6 ± 0.8%), *Blautia* (1.6 ± 0.4%)*, Faecalibacterium* (0.7 ± 0.2%), and *Prevotella* (0.1 ± 0.1%) were present at lower abundances.

### 3.3. Reduction of bacterial concentration by adapting ileal nutritional medium

**Figure 5:**
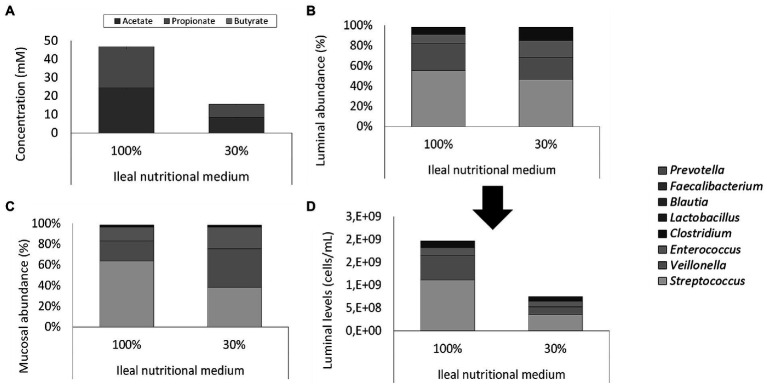
**(A)** Average SCFA concentrations (mM) in steady-state ileum simulations being fed with two types of ileum nutritional medium (100% vs. 30% content) after inoculation of a synthetic consortium under reference conditions. **(B)** Proportional and **(D)** quantitative luminal genus level microbial composition of steady-state ileum simulations being fed with two different ileal nutritional media (100% vs. 30%) as determined *via* 16S rRNA gene targeted Illumina sequencing. The quantitative composition was obtained through of 16S rRNA gene targeted Illumina sequencing and flow cytometry data. of steady-state ileum simulations being fed with two different ileum nutritional media (100% vs. 30% sugar content). **(C)** Proportional mucosal microbial composition in two ileum simulations receiving different types of nutritional medium (100% vs. 30%) as determined by 16S rRNA gene targeted Illumina sequencing. The reference *Lactobacillus* is considered to include both the genera *Limosilactobacillus* and *Ligolactobacillus.*

Total cell concentrations were estimated from qPCR measurements based on a post-hoc comparison between flow cytometry and qPCR analyses. Based on this correlation, qPCR analysis during the screening experiment pointed out that levels above 10^9^ cells/ml in the ileum simulations were obtained, which was considered too high versus the *in vivo* situation where levels in the range of 10^8^ cells/ml have been reported (Booijink et al., 2007). Therefore, with the aim of reducing the bacterial load in the ileum simulation, adapted ileal nutritional medium (30%) was compared to the initial ileal nutritional medium (100%) in two identically configured vessels. Importantly, bacterial cell quantification through flow cytometry demonstrated an approximately 3-fold reduction in total bacterial density upon administration of the adapted ileal nutritional medium (2.53 and 7.58 * 10^8^ cells/ml at the beginning and end of the feeding cycle, respectively) as compared to the initial ileal nutritional medium (6.65 and 19.95 * 10^8^ cells/ml at beginning and end of the feeding cycle, respectively; Figure 5D).

This decreased cell density did not alter microbial activity or community composition. Indeed, while the adapted medium (30%) significantly (*p* = 2 * 10^−8^) reduced total SCFA concentration, it did not alter the acetate/propionate/butyrate ratio (approximately 55/45/0; Figure 5A). Lactate was significantly (*p* < 10^−5^) higher – but still minimal – in the adapted medium (0.025 ± 0.003 mM) than the initial medium (0.007 ± 0.002 mM). 16S rRNA gene targeted Illumina sequencing revealed a similar microbial composition in both the luminal and mucosal regions between both nutritional media (Figures 5B,C). Lowered nutrient levels resulted in a mild proportional increase of *Clostridium* (12.5% vs. 6.8%) and *Enterococcus* (16.7% vs. 8.7%) at the expense of *Streptococcus* (46.7% vs. 55.6%) and *Veillonella* (21.8% vs. 26.7%). Hence, where streptococci and *Veillonellae* accounted for more than 80% of the total community in the vessel receiving the 100% nutritional medium, these taxonomic groups represented approximately 65% of the community in the vessel receiving the adapted ileal nutritional medium (30%). Furthermore, *Lactobacillus* (0.2% vs. 0.2%), *Blautia* (0.1% vs. 0.1%), *Faecalibacterium* (<0.1% vs. *<*0.1%) and *Prevotella* (<0.1% vs. <0.1%) were not impacted. Together, genera comprised in the synthetic consortium accounted for more than 98% of the simulated community (98.0 and 98.2% for adjusted and initial nutritional medium, respectively). The missing approximately 2% relative abundance is represented by *Lactococcus* (1.2% vs. 1.3% for adjusted and initial nutritional medium, respectively), *Hungatella* (0.20% vs. 0.15%, for adjusted and initial nutritional medium, respectively) and a myriad of low-abundant genera (<0.1%; data not shown).

### 3.4. Integration of ileum simulation in M-SHIME^®^ model

During the final experiment, the ileal simulation was integrated into the established M-SHIME® model which simulates colonic microbiota. For this, two arms for each of two donors were run in parallel. Per donor, one arm comprised of an ileum, proximal colon, and distal colon, whereas the second arm consisted of a proximal and distal colon only.

**Figure 6:**
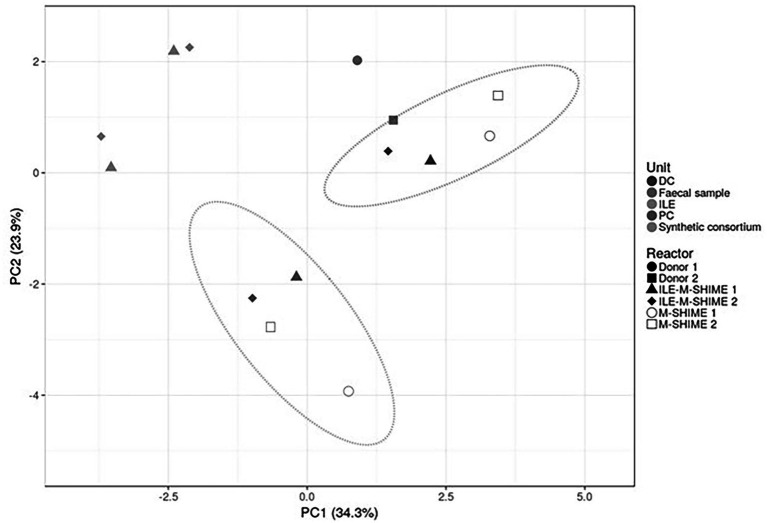
Principal component analysis (PCA) of microbiota based on proportional 16S rRNA gene targeted Illumina sequencing data. A total of 16 taxonomic families was considered, i.e., the 15 most abundant families supplemented with taxonomic families representing consortium genera not being part of the top 15 most abundant families. Percentage values at the axes indicate contribution of the principal components to the explanation of total variance in the data set. The impact of integrating an ileum before the PC and DC (ILE-M-SHIME®) was compared to the standard M-SHIME® for two donors.

**Figure 7:**
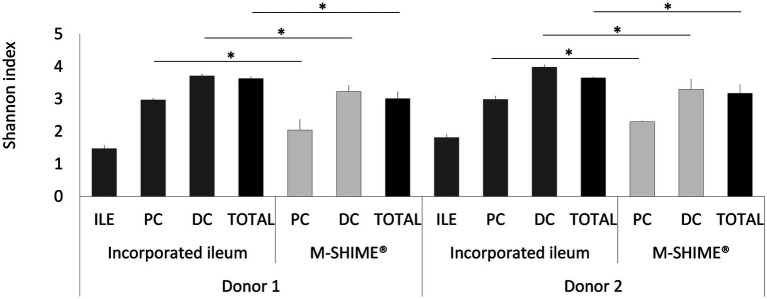
Shannon diversity indices based on (OUT’s) in ileum simulation, and colon vessels whether or not preceded by an ileum simulation (ILE-M-SHIME® vs. M-SHIME®) as calculated from 16S rRNA gene targeted Illumina sequencing data. Diversity estimates were determined for separate vessels and total reactor units for two donors at three different timepoints (days 11, 13, and 14 after inoculation). Total diversity indices that were significantly different from each other, are indicated with an asterisk (*). Only OTU’s with >25 reads were considered.

As illustrated by a PCA, based on the 15 most abundant families across the lumen of all reactors combined with families to which the genera of the ileal consortium belong but are not within the top 15 most abundant families, colon-region specific communities colonize the M-SHIME® model (Figure 6). Integration of an ileum simulation in the M-SHIME® maintained this colon-region specificity, thereby indicating that a preceding ileum does not disturb subsequent simulated colonic communities. Moreover, inclusion of the ileum microbiota contributed to an increased diversity (Figure 7) as well as an increased species richness (data not shown) in the *in vitro* gut model for both donors, reflecting more what has been reported happing *in vivo*. Furthermore, it was observed that distal colons more closely resembled the faecal inocula when preceded by an ileum as compared to distal colons without preceding ileum (Figure 6).

**Figure 8:**
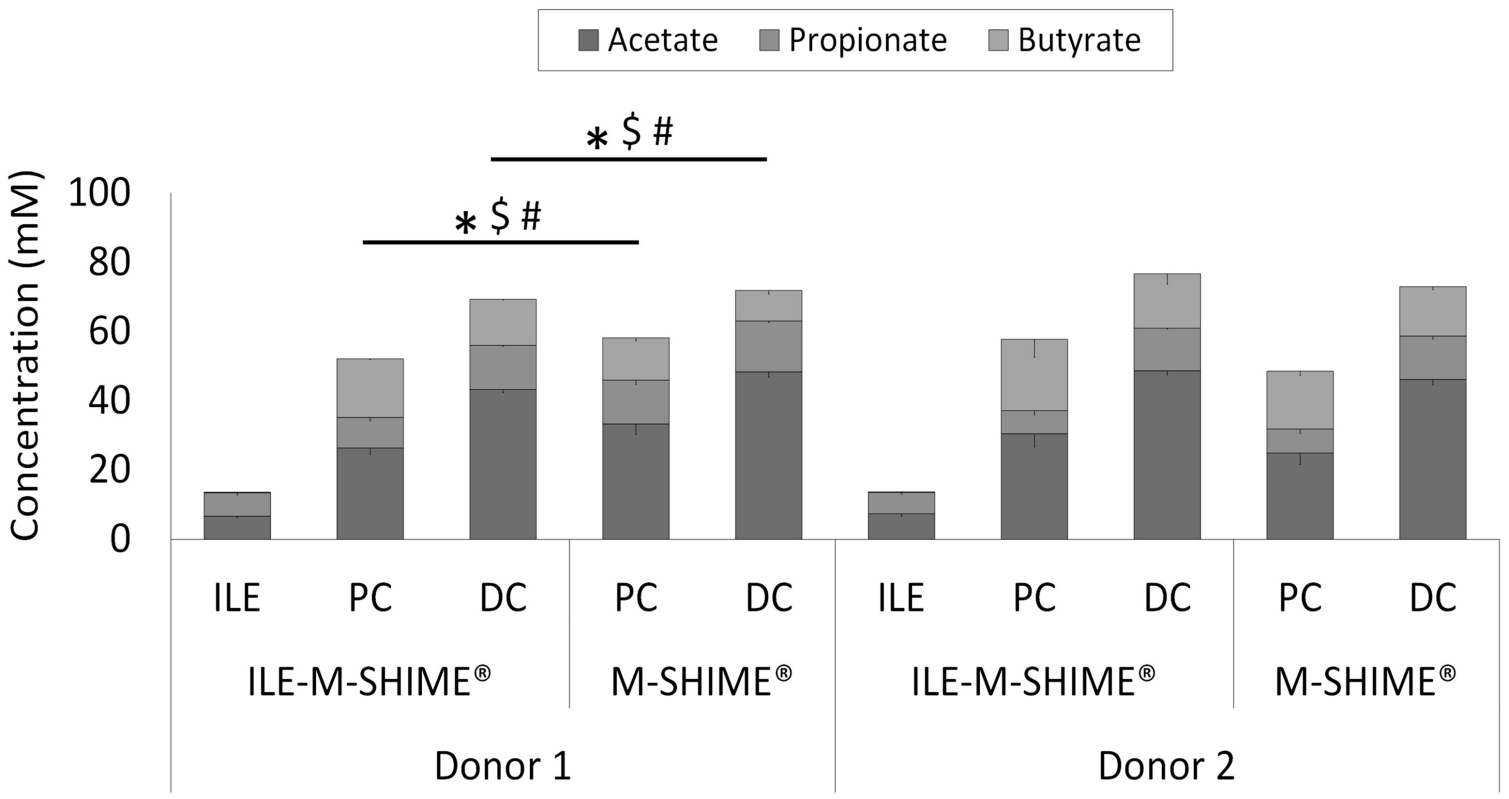
Average SCFA concentrations (mM) in steady-state colon vessels whether or not preceded by an ileum simulation (ILE-M-SHIME® vs. M-SHIME®, respectively). Symbols indicate significant differences in acetate (*), propionate ($) or butyrate (#) between corresponding region simulations of the same donor. ILE = ileum; PC = proximal colon; DC = distal colon.

Although integration of an ileum reactor increased initial lactate levels in the proximal (0.84 ± 0.14 mM to 2.20 ± 0.06 mM) and distal (0.22 ± 0.04 mM to 0.32 ± 0.01 mM) colons, lactate was more rapidly consumed in ileum-preceded colon reactors. SCFA analysis confirmed both ileum simulations to show nearly identical SCFA profiles (Figure 8). The total SCFA concentrations were 13.5 ± 0.7 mM and 13.9 ± 0.5 mM, and the acetate/propionate/butyrate ratios were 50/49/1 and 54/45/2 in the ileum vessels of reactor units 1 and 2, respectively. Incorporation of an ileum vessel did not impact the total SCFA concentration in proximal (54.8 ± 3.8 mM vs. 53.1 ± 6.3 mM with or without preceding ileum, respectively) or distal colon vessels (73.0 ± 4.2 mM vs. 72.5 ± 2.1 mM with and without preceding ileum, respectively). However, preceding ileum simulations impacted the proportional SCFA profiles in the subsequent colon compartments. For donor 1, the preceding ileum increased butyrate concentration in both colon vessels at the expense of acetate and propionate (SCFA ratios changed from 58/22/21 to 51/17/32 for the PC, and from 67/21/12 to 62/18/19 for the DC). Nonetheless, for donor 2, SCFA ratios remained constant (from 52/14/34 to 54/11/35 for the PC, and from 63/17/20 to 64/16/20 for the DC).

**Figure 9:**
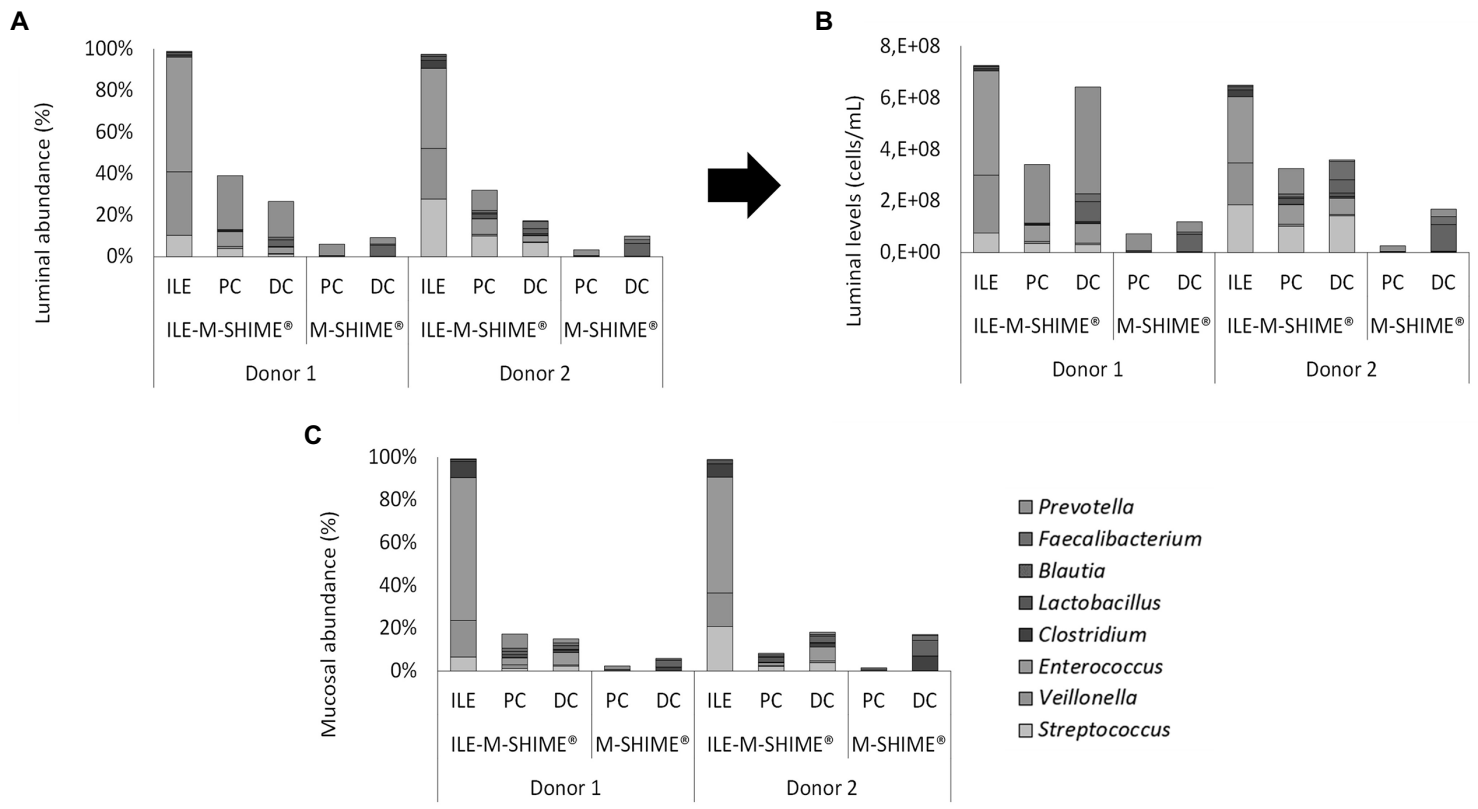
**(A)** Proportional and **(B)** quantitative composition of the luminal and mucosal **(C)** community in steady-state colon simulations whether or not preceded by an ileum simulation (ILE-M-SHIME® vs. M-SHIME®, respectively) as determined by 16S rRNA gene targeted Illumina sequencing. Ileum vessels were inoculated with a synthetic consortium under reference conditions. Quantitative compositions were obtained through combination of 16S rRNA gene targeted Illumina with flow cytometry data. Only genera included in the synthetic consortium are presented. The reference *Lactobacillus* is considered to include both the genera *Limosilactobacillus* and *Ligolactobacillus*.

Regarding microbial composition, steady-state communities in the lumen and mucus of both ileum simulations consisted nearly entirely of genera included in the synthetic consortium (98.8 ± 0.4% and 99.3 ± 0.2%, and 97.3 ± 0.3% and 98.8 ± 0.2% for the lumen and mucus of ileum vessel of unit 1 and 2, respectively), indicating absence of colonization by contaminating strains (Figures 9A–C). In the ileum vessels of reactor units 1 and 2, stable luminal communities were dominated by the genera *Veillonella* (30.6 ± 4.6% and 24.3 ± 6.8%, respectively), *Streptococcus* (13.0 ± 5.0% and 27.8 ± 7.0%, respectively), and *Enterococcus* (55.1 ± 1.6% and 38.5 ± 1.1%, respectively).

Integration of an ileum simulation into the M-SHIME® technology did not affect bacterial densities in subsequent proximal and distal colon reactors. Moreover, incorporation of the ileum simulation did not majorly affect the luminal bacterial community composition in neither the proximal nor distal colon (Supplementary Table S1). Minor changes included, for donor 1, a reduction in Bacteroidaceae (PC), Selenomonadaceae (PC), Synergistaceae (DC), and Akkermansiaceae (DC), while the levels of Enterococcaceae (PC and DC) and Streptococcaceae (PC and DC) were increased. Although not significantly, Ruminococcaceae levels were increased in the PC upon incorporation of an ileum simulation. For donor 2, Streptococcaceae and Enterococcaceae were increased in both the PC and DC, while the Ruminococcaceae were significantly increased in the PC and significantly decreased in the DC. Changes in mucosal community compositions were less pronounced.

## 4. Discussion

The present study focused on the development of a stable and reproducible long-term *in vitro* model of the ileal microbiota that is representative for the *in vivo* situation. After its successful development as a stand-alone model, the simulation was integrated in the M-SHIME® model that simulated up till now only the colonic microbiota, thereby further increasing the relevance of the latter model.

First, the established simulated ileal bacterial community after 2 weeks of incubation under reference conditions, involving amongst other factors inoculation with a synthetic consortium, application of a short retention time (4 h) and a nutritional medium rich in simple sugars, was relevant for the *in vivo* situation with the genera *Streptococcus*, *Veillonella*, and *Enterococcus* prevailing in the model. Enrichment of these genera is in line with literature regarding microbial composition of ileostomy effluent which has often been used as a *proxy* for investigating the ileal community (Collado et al., 2007; Booijink et al., 2010; van den Bogert et al., 2011, 2013; Zoetendal et al., 2012; Dlugosz et al., 2015; El Aidy et al., 2015; Chung et al., 2016). Abundances of selected consortium members well-above detection limit after 2 weeks of incubation confirmed the relevance of the applied environmental conditions. The observation that increasing the retention time to 8 h impaired colonization of *Veillonella* and *Streptococcus* members, shows the complexity of identifying the correct environmental conditions. It is hypothesized that this community shift can be attributed to prolonged metabolism of less favorable sugars when retention time is increased to 8 h, thereby promoting colonization of specific consortium members and potentially stimulating build-up of inhibitory metabolites and associated product inhibition. This would be in contrast with the reference conditions where a retention time of 4 h is potentially too short for fermentation of less favorable sugars, thereby stimulating consortium members that thrive on the most-favored carbon source (e.g., glucose). On the other hand, the reference condition did not prevent colonization of colon-like microbial groups upon inoculation with a faecal sample or ileostomy effluent, suggesting further potential for finetuning the environmental conditions by, e.g., inclusion of a fill-and-draw mechanism or incorporating colon-to-ileum reflux simulations as attempted recently (Roussel et al., 2020). Nonetheless, the current model enabled the establishment and maintenance of a representative bacterial community in terms of composition and, importantly, quantity.

The microbial composition showed an ileum-relevant microbial activity. More specific, upon entrance of fresh nutritional medium, lactate levels rapidly increased during the first 30 min, after which acetate and propionate levels increased while lactate was consumed, thus suggesting bacterial cross-feeding interactions. The initial boost in lactate production was likely attributed to the *Streptococcus*, *Enterococcus*, and *Lactobacillus* species which are known to produce lactate upon consumption of simple sugars (Hoefman et al., 2012; Lee and Lee, 2018). In turn, successive cross-feeding of lactate into acetate and propionate was likely ascribed to high abundances of *Veillonella* species (Distler and Kröncke, 1981; Arif et al., 2008). The relatively short retention time (4 h) is thought to prevent substantial butyrate production in the ileum, as is supported by low abundances of slow-growing *Faecalibacterium* and *Clostridium*. Increased retention time strongly increased lactate production and prevented cross-feeding interactions. The latter might be explained by a prolonged utilization of less-favorable carbohydrates upon glucose depletion. Genera such as *Enterococcus* and *Lactobacillus* are known to be more flexible in carbohydrate fermentation, thereby being favored by an increased retention time. Higher abundances of these genera could result in a boosted production of inhibiting metabolites such as lactate and ethanol, which might hamper the proliferation of, e.g., *Streptococcus* and *Veillonella.* Nonetheless, further investigation of this hypothesis is crucial for unraveling the mechanisms through which the increased retention time impacts the simulated community. In turn, inoculation with ileostomy effluent or faecal slurry prevented substantial buildup of lactate concentrations, which is likely explained by higher cross-feeding efficiency and lower lactate-producing capacity of the colon-like communities in these vessels. Furthermore, the established community under selected reference conditions and the condition with increased retention time only partially deconjugated the bile salts TCA, TDCA, GCA, and GDCA in the model. *In vivo*, most of the bile salts are reabsorbed in the ileum (enterohepatic circulation) but some are biotransformed by the gut microbiota. This biotransformation involves a first step of deconjugation (ileum), after which the deconjugated bile acid can be dehydroxylated and/or reconjugated in the colon. These bioconversions facilitate transport and improve functionalities and signaling properties of the bile salts. Hence, what observed corresponds to what happens *in vivo*, where unabsorbed bile salts are converted and/or deconjugated only partially in the ileum region (Hofmann, 1999; Martinez-Augustin and Sanchez de Medina, 2008). Opposed thereto, conditions inoculated with ileostomy effluent or faecal slurry more closely resembled the bile salt metabolism as observed in the control colon, i.e., nearly complete deconjugation and conversion of primary bile salts. Although not confirmed in the present study, these observations are likely to be linked to a limited presence of genes encoding for bile salt hydrolase and 7-α-dehydroxylase in the gene pool of the established ileum community as compared to the colon community. Overall, the optimized ileum simulation allowed mimicking of metabolic cross-feeding interactions between key members of the consortium while revealing a representative bile salt metabolism, thus suggesting that the model might be a useful tool in future research to study the microbial metabolite fluxes in the highly dynamic small intestinal environment (Seekatz et al., 2019).

The selected simulation strategy allowed the recreation of a highly reproducible microbial composition and activity within independent experiments. Nevertheless, between different experiments, minor shifts in relative bacterial profiles were detected although these did not appear to impact metabolic functionality. For instance, the genera *Streptococcus*, *Enterococcus*, and *Veillonella* accounted for approximately 85% of the ileal community (41, 11, and 31%, respectively) during the reproducibility experiment, while during the final experiment, combined relative abundances of the *Streptococcus*, *Enterococcus*, and *Veillonella* genera accounted for roughly 95% with *Streptococcus* being less abundant than *Enterococcus* (on average, 19, 47, and 28%, respectively). It is hypothesized that these compositional differences could follow from the reduced sugar concentration in the nutritional medium which was only 30% of the sugar content from the reproducibility experiment. In particular, reduced glucose administration might have expedited the diauxic shift towards less favorable sugars, thereby relatively stimulating consortium members that more efficiently metabolize such less-favorable sugars (e.g., *Enterococcus*). Nonetheless, further research is necessary in order to confirm these hypotheses.

Reducing bacterial densities in the ileum simulation increased biological relevance of the simulation and enabled integration of the ileum into the M-SHIME® technology. Feeding the ileum vessel with adapted ileal nutritional medium (30%) reduced the total bacterial load 3-fold as compared to the model fed with the initial ileal nutritional medium (100%). On average, the bacterial load comprised 2.53 and 7.58 * 10^8^ cells/ml (at beginning and end of the feeding cycle, respectively) which is slightly higher than the *in vivo* levels of 10^7^–10^8^ cells/ml being reported (Kastl et al., 2020). However, it was already hypothesized that actual *in vivo* levels are substantially higher than what is being reported due to the bias of the fasted state of patients upon sampling (Zoetendal et al., 2012; El Aidy et al., 2015; Villmones et al., 2018, 2020). This is illustrated by the observation of higher bacterial densities in samples from ileostomists after intake of carbohydrate-rich food. As such, it was hypothesized that ileal bacterial levels might fluctuate locally upon passage of nutrients, with bacterial loads increasing upon prolonged interaction between nutrient rich chyme and bacteria. Nonetheless, the optimized bacterial load in the current model allows studying metabolic shifts and community dynamics, whereas lower bacterial levels would likely result in shifts that are too close to the limit of quantification to allow proper investigation of induced changes. Overall, after optimization of the bacterial load, the latter more closely mimicked *in vivo* levels of 10^7^–10^8^ cells/g and facilitated integration of the ileum simulation into the M-SHIME® technology.

Although the M-SHIME® model has been thoroughly validated and is known to reliably represent *in vivo* communities of both proximal and distal colon communities (Van den Abbeele et al., 2010), incorporation of the ileum model into the M-SHIME® seemed to further increase its biological relevance. First, inclusion of a bacterial community resembling the ileal microbiota according to criteria elaborated above, as such already mimics the *in vivo* situation more closely. Second, a preceding ileum tended to increase the observed number of species and diversity. Presumably, the preceding ileum incubation provides metabolites and substrates to the colon regions that support growth of otherwise washed-out species. Despite high recovery rates of bacterial strains between faecal samples and stabilized communities using the M-SHIME®, some taxonomic groups appear to be less effective at colonizing the conventional SHIME® model. For instance, Ruminococcaceae are often targeted as a health marker since they encompass health-promoting species like *F. prausnitzii*. However, Ruminococcaceae are typically underrepresented in especially the proximal (or ascending) colon of the M-SHIME® as compared to the faecal sample used as inoculum (Van den Abbeele et al., 2010). Inclusion of the ileum model increased the abundance of Ruminococcaceae in the proximal colon of both donor 1 and 2 as compared to the respective simulations without preceding ileum microbiota. This could be attributed to the influx of specific metabolic products (e.g., acetate) originating from the ileum, thereby creating a stimulating niche for Ruminococcaceae. Such metabolic interactions might have contributed to the observation that samples derived from the distal colon of the SHIME® with preceding ileum clustered more closely to the faecal inocula than samples from the distal colon from the SHIME® without preceding ileum. Development of the ILE-M-SHIME® model can therefore further increase the relevance of long-term *in vitro* simulations of the colonic microbiota using the SHIME® technology.

## 5. Conclusion

In conclusion, the present study describes the development of a stable, reproducible, and representative long-term *in vitro* simulation of a human ileal microbial community that can be incorporated in the established M-SHIME® technology for the human colonic microbiota. Further studies will be needed to elucidate the role of this specific microbial community in influencing digestibility, drug stability and nutrient absorption. Although not replacing *in vivo* experimental studies, it is suggested that this model can serve as a primary screening method to reveal insights into the ileal microbiota, its interaction with therapeutics, and stimulate development of more efficient treatment methods.

## Data availability statement

The data presented in the study are deposited in the NCBI repository, accession number PRJNA940490 (link: https://www.ncbi.nlm.nih.gov/bioproject/PRJNA940490).

## Author contributions

SD, FM, Bv, MM, TV, MK, and PV developed the study concept. SD carried out the experiment and collected the data, except for the sequencing data that were generated by WP, Bv, and EK. SD, FM, and PA evaluated the data and wrote the manuscript, which was then proofread and approved by Bv, MM, TW, and MK. All authors contributed to the article and approved the submitted version.

## Funding

This work was financially supported by the Eurostars project Promise (E12091).

## Conflict of interest

FM, MM, and TW are employees of ProDigest, that provides services to other companies using the model developed in this study. SD and PA were employees of ProDigest when this project was conducted. During the current project WP, Bv, and EK were employed by BaseClear, the company providing NGS services throughout the study.

The remaining authors declare that the research was conducted in the absence of any commercial or financial relationships that could be construed as a potential conflict of interest.

## Publisher’s note

All claims expressed in this article are solely those of the authors and do not necessarily represent those of their affiliated organizations, or those of the publisher, the editors and the reviewers. Any product that may be evaluated in this article, or claim that may be made by its manufacturer, is not guaranteed or endorsed by the publisher.

## Abbreviations

BCFA: Branched-chain fatty acid
GIT: Gastrointestinal tract
SCFA: Short-chain fatty acid
M-SHIME: Mucosal Simulator of the Human Intestinal Microbial Ecosystem
ILE: Ileum
PC: Proximal colon
DC: Distal colon
SC: Synthetic consortium
FS: Faecal sample
IE: Ileostomy effluent
TSB: Tryptic soy broth
MRS: de Man, Rogosa and Sharpe medium
RCM: Reinforced clostridial medium
RT: Retention time
REF: Reference conditions
TCA: Taurocholic acid
GCA: Glycocholic acid
TDCA: Taurodeoxycholic acid
GDCA: Glycodeoxycholic acid
OTU: Operational taxonomic unit
LOQ: Limit of quantification
DAD: Diode array detector
FDR: False discovery rate
PTFE: Polytetrafluorethene
RP-HPLC: Reversed phase high pressure liquid chromatography
PCA: Principal component analysis
BSH: Bile salt hydrolase
