# Supplementary material for: Development of a reproducible small intestinal microbiota model and its integration into the SHIME®-system, a dynamic in vitro gut model
Source: Front Microbiol. 2023 Mar 17;13:1054061. doi: 10.3389/fmicb.2022.1054061 (PMC10063983; doi:10.3389/fmicb.2022.1054061)
Supplement: Supplementary file 1 [file Data_Sheet_1.docx]

Supplementary Material

**Supplementary figure S1: (A)** Proportional and **(B)** quantitative composition of the luminal and mucosal **(D)** community in steady-state colon simulations whether or not preceded by an ileum simulation (ILE-M-SHIME^®^ *vs.* M-SHIME^®^, respectively) as determined by 16S rRNA gene targeted Illumina sequencing. Ileum vessels were inoculated with a synthetic consortium under reference conditions. Quantitative compositions were obtained through combination of 16S rRNA gene targeted Illumina with flowcytometry data. Only the 15 most abundant families are included.

**Supplementary figure S2:** Temporal profile of SCFA and lactate concentrations (mM) in reactors comprising a steady-state community upon administration of fresh nutritional medium and sequential liquid transfers: **(A)** ileum reactor of unit 1 inoculated with synthetic consortium; **(B)** ileum reactor of unit 2 inoculated with synthetic consortium; **(C)** proximal colon inoculated with faecal slurry of donor 1; **(D)** proximal colon inoculated with faecal slurry of donor 2; **(E)** distal colon inoculated with faecal slurry of donor 1; **(F)** distal colon inoculated with faecal slurry of donor 2. Full lines represent metabolite profiles within the ILE-M-SHIME^®^, dashed lines represent metabolite profiles within the M-SHIME^®^.

**Supplementary table S1. Average absolute levels (^10^log(16S rRNA copies/mL)) of 10 different taxonomic groups (as determined with group-specific qPCR protocols) that, according to literature, colonize the small intestine and/or colon, in a faecal slurry and ileostomy effluent (20:100 (w:v)).** The value of a sample that contained the highest level of a given taxonomic group is indicated in bold. LOQ = 3.31 ^10^log(16S rRNA copies/mL).

| **Region of colonization according to literature** | **Microbial group** | **Faecal slurry** | **Ileostomy effluent** |
| --- | --- | --- | --- |
| **Small intestine** | **Veillonellaceae** | <LOQ | **7.10** |
|  | **Streptococcaceae** | 5.88 | **7.52** |
|  | **Enterococcaceae** | 5.75 | **6.23** |
|  | **Lactobacillaceae** | <LOQ | **5.28** |
| **Small intestine - colon** | **Enterobacteriaceae** | **5.79** | 5.35 |
| **Colon** | **Akkermansiaceae** | **5.11** | <LOQ |
|  | **Bifidobacteriaceae** | **7.08** | 4.79 |
|  | **Bacteroidetes** | **7.92** | <LOQ |
|  | ***C. coccoides / E. rectale*** | **8.57** | 5.00 |
|  | ***F. prausnitzii*** | **9.46** | <LOQ |

**Supplementary table S2: Average differences in absolute levels (^10^log(16S rRNA copies/mL)) of 15 most abundant taxonomic families (as determined with 16S rRNA gene targeted Illumina sequencing and flowcytometry) between colon communities in an M-SHIME^®^ configuration with or without integrated ileum simulation.** Significant differences are indicated in bold. ILE = ileum; PC = proximal colon; DC = distal colon.

|  |  | **Lumen** | | | | | **Mucus** | | | | |
| --- | --- | --- | --- | --- | --- | --- | --- | --- | --- | --- | --- |
|  |  | **ILE** | **ILE-M-SHIME^®^**  ***vs.***  **M-SHIME^®^** | | | | **ILE** | **ILE-M-SHIME^®^**  ***vs.***  **M-SHIME^®^** | | | |
|  |  |  | **Donor 1** | | **Donor 2** | | **ILE** | **Donor 1** | | **Donor 2** | |
| **Phylum** | **Family** | **U1 *vs.* U2** | **PC** | **DC** | **PC** | **DC** | **U1 *vs.* U2** | **PC** | **DC** | **PC** | **DC** |
| Actinobacteria | Bifidobacteriaceae | -0.81 | -0.06 | -0.10 | 0.19 | -0.17 | -0.15 | -0.03 | -0.04 | 0.02 | -0.02 |
| Bacteroidetes | Bacteroidaceae | -0.33 | **-0.98** | -0.03 | -1.31 | -0.21 | -0.01 | **-0.24** | -0.07 | -0.11 | -0.05 |
|  | Porphyromonadaceae | 0.00 | -0.34 | 0.02 | -0.15 | -0.17 | **0.00** | 0.00 | -0.02 | 0.00 | 0.00 |
|  | Prevotellaceae | -0.01 | 0.75 | 0.84 | 0.54 | -0.90 | 0.00 | 0.13 | 0.06 | 0.04 | 0.00 |
| Firmicutes | Enterococcaceae | 0.28 | **2.31** | **2.31** | **2.52** | **1.83** | **0.29** | **0.04** | **0.05** | 0.03 | **0.04** |
|  | Streptococcaceae | **-0.37** | **1.75** | **1.78** | **2.80** | **2.25** | **-0.08** | 0.03 | 0.02 | 0.04 | 0.04 |
|  | Clostridiaceae | -0.22 | 0.23 | 0.31 | 0.29 | -0.15 | 0.05 | 0.00 | 0.00 | 0.00 | -0.01 |
|  | Lachnospiraceae | -1.36 | 0.12 | 0.14 | 0.31 | -0.15 | -0.02 | 0.02 | 0.01 | -0.02 | -0.02 |
|  | Ruminococcaceae | -0.74 | 0.49 | -0.05 | **1.93** | **-0.23** | 0.00 | 0.02 | 0.00 | **0.01** | 0.04 |
|  | Acidaminococcaceae | -1.07 | -0.10 | 0.20 | 0.20 | -0.12 | -0.05 | 0.01 | 0.02 | 0.03 | 0.01 |
|  | Selenomonadaceae | -0.23 | **-2.44** | -0.21 | 0.19 | 0.01 | -0.01 | -0.07 | 0.00 | 0.01 | 0.00 |
|  | Veillonellaceae | 0.31 | -0.01 | 0.29 | -0.01 | -0.50 | 0.01 | 0.01 | 0.02 | -0.06 | 0.00 |
| Proteobacteria | Desulfovibrionaceae | 0.00 | -0.65 | -0.12 | -0.52 | -0.20 | 0.00 | 0.00 | -0.01 | 0.00 | 0.00 |
| Synergistetes | Synergistaceae | 0.00 | 0.18 | **-3.08** | 0.00 | -0.19 | 0.00 | 0.00 | -0.05 | 0.00 | 0.02 |
| Verrucomicrobia | Akkermansiaceae | 0.00 | 0.00 | **-1.95** | 0.00 | -0.80 | 0.00 | 0.00 | 0.00 | 0.00 | -0.01 |
